# Editorial: Analytical chemistry of rare earth elements (REEs)

**DOI:** 10.3389/fchem.2022.1127842

**Published:** 2023-01-10

**Authors:** Yanbei Zhu, Giovanni Mongelli, Rosa Sinisi, Yong-Hyeon Yim

**Affiliations:** ^1^ National Metrology Institute of Japan (NMIJ), National Institute of Advanced Industrial Science and Technology (AIST), Tsukuba, Japan; ^2^ Department of Sciences, University of Basilicata, Potenza, Italy; ^3^ National Research Council of Italy, Institute of Methodologies for Environmental Analysis (CNR-IMAA), Tito Scalo, Potenza, Italy; ^4^ Korea Research Institute of Standards and Science (KRISS), Daejeon, South Korea

**Keywords:** rare earth elements, analysis, SIMS, ICP-MS, gravimetric analysis

Rare earth elements (REEs)—including Y, Sc, and lanthanides—are a group of elements attracting interests from various research fields due to general similarities and some variation in physical and chemical properties. On the one hand, REEs are treated as a set of elements tracing the chemical processes in geochemistry, marine chemistry, and environmental chemistry. On the other hand, individual REEs, with their unique physical properties, are indispensable materials in modern industry, especially for the semiconductor industry. There are multiple Research Topic published since 2020 treating REEs as functional materials (Bunzli et al., 2020; Economou-Eliopoulos et al., 2020; Li, 2020; Zhang H.J. and Zhang H., 2022) and resources (Han, 2021; Kynicky et al., 2021; Tshentu and Parajuli, 2022). The present Research Topic aims to highlight the analytical chemistry of REEs, which contributes to the development and the application of REEs in other research fields.

Four original works on analytical chemistry of REEs are collected in this Research Topic, covering high lateral resolution secondary ion mass spectrometry (NanoSIMS), inductively coupled plasma tandem quadrupole mass spectrometry (ICP-QMS/QMS), gravimetric analysis, and multi-collector inductively coupled mass spectrometry (MC-ICP-MS).

Shi et al. reported a method to analyze all REEs in silicate glasses and zircon minerals by NanoSIMS. A crater with a diameter of 7–8 μm was obtained on the sample. Separation of heavy REE from oxide of light REE was achieved at a high mass resolving power of 9,400at 10% peak height. The results of REEs in NIST SRM610 glass showed sensitivities from 3 cps/ppm/nA of Lu to 13 cps/ppm/nA of Eu. The method was applied to the determination of REEs in AS3, QGNG, and Torihama zircons, providing satisfied results in comparison to glass standard and reported data. Partition coefficients of REEs between silicate melt and zircon were obtained by combination of Torihama REE data with the whole rock data.

Zhu investigated nitrous oxide (N_2_O) as the reaction gas for measuring REEs by ICP-QMS/QMS. It was found that the yields of ^*m*^M^16^O^+^ for Eu and Yb were apparently improved by using N_2_O as the reaction gas. Taking advantage of this improvement, high sensitivity measurement of the whole set of REEs including Eu and Yb was achieved, with a typical sensitivity for mono-isotopic REEs at 300,000 CPS mL/ng. The use of N_2_O as the reaction gas was also effective to suppress Ba related spectral interferences with the measurement of Eu, resulting in free-of-mathematic-correction measurement of Eu in natural sample.

Miura and Wada applied gravimetric analysis to evaluate the purity of high-purity La_2_O_3_ by stepwise conversions of the weighing forms, where La in the sample was respectively converted to weighing forms of La oxalate, La_2_O_3_, and La_2_(SO_4_)_3_, which were used to evaluate the stoichiometry for accurate gravimetric determination. Inductively coupled plasma optical emission spectrometry (ICP-OES) was used to determine the losses of La during the filtration process. The observed La_2_(SO_4_)_3_ was consistent with the theoretical composition based on the observed mass ratio of La_2_(SO_4_)_3_/La_2_O_3_. The purity value for the original La_2_O_3_ sample was 99.977% ± .057%, which combined the gravimetric analysis using the precipitation from the homogeneous solution and verification of the weighing forms for La compounds. Impurities in the high-purity La_2_O_3_ were determined by ICP-QMS/QMS.

By using 2-hydroxyisobutyric acid (HIBA) for group separation REEs prior to their measurements by MC-ICP-MS, Lee and Ko developed a method for accurate and precise determination of REEs (particularly, heavy REEs) concentrations in geological materials including natural waters. By using a cation exchange column (AG50W-X8 200–400 mesh) with HIBA, REEs were separated to three groups, i.e. light REEs (LREE, La-Ce-Pr-Nd), middle REEs (MREE, Sm-Eu-Gd-Tb) and heavy REEs (HREE, Dy- Ho-Er-Tm-Yb-Lu). Recovery rates of most REEs in natural water sample and rock sample exceeded 98% and 95%, respectively. This method permits free-of-spectral-interference measurement of the whole set of REEs (particularly, HREE) in geological materials, contributing to correct interpretation of geochemical implications of REEs in geological system.
